# Whole lung lavage and GM-CSF use for pulmonary alveolar proteinosis in an infant with lysinuric protein intolerance: a case report

**DOI:** 10.1186/s13052-024-01677-y

**Published:** 2024-06-03

**Authors:** Eszter Vojcek, Dóra Krikovszky, Csaba Lódi, Lajos Kovács, János Schnur, Attila J. Szabó

**Affiliations:** 1 Pediatric Center, MTA Center of Excellence, Semmelweis University, Bókay J. u. 53-54, Budapest, 1083 Hungary; 2 Heim Pál Children’s Hospital, Budapest, Hungary

**Keywords:** Lysinuric protein intolerance, Pulmonary alveolar proteinosis, Whole lung lavage, Granulocyte-Macrophage colony stimulating factor, Veno-venous ECMO, Case report

## Abstract

**Background:**

Lysinuric protein intolerance (LPI) is a multi-organ metabolic disorder characterized by the imbalance in absorption and excretion of cationic amino acids like lysine, ornithine and arginine. Infants with LPI typically present with recurrent vomiting, poor growth, interstitial lung disease or renal impairment. The early onset of pulmonary alveolar proteinosis (PAP) has been reported to be associated with a severe form of LPI. Treatment of PAP most commonly consists of whole-lung lavage (WLL) and in autoimmune PAP, granulocyte-macrophage colony stimulating factor (GM-CSF) administration. Nevertheless, GM-CSF therapy in LPI-associated PAP has not been scientifically justified.

**Case presentation:**

We describe the case of an 8-month-old infant presenting with respiratory failure due to LPI associated with PAP, who was twice treated with WLL; firstly, while on veno-venous ECMO assistance and then by the use of a selective bronchial blocker. After the two treatments with WLL, she was weaned from daytime respiratory support while on initially subcutaneous, then on inhaled GM-CSF therapy.

**Conclusions:**

This case supports the notion that GM-CSF therapy might be of benefit in patients with LPI-associated PAP. Further studies are needed to clarify the exact mechanism of GM-CSF in patients with LPI-associated PAP.

**Supplementary Information:**

The online version contains supplementary material available at 10.1186/s13052-024-01677-y.

## Background

Lysinuric protein intolerance (LPI) is an autosomal recessive disorder first described by Perheentupa and Visakorpi in 1965 [1]. The highest prevalence of LPI is reported in Finland affecting approximately 1 in 60.000 births [2]. It is a multi-organ congenital metabolic disorder leading to faulty transport of cationic amino acids like lysine, ornithine and arginine of the epithelial cells, mainly at the basolateral membrane of the lungs, kidneys and intestine [3]. Infants with LPI typically present with recurrent vomiting, failure to thrive and muscular hypotonia [4]. Additionally, poor growth, hepatosplenomegaly, osteoporosis, interstitial lung diseases and renal impairment evolve over time [5].

The early onset of pulmonary alveolar proteinosis (PAP) has been reported to be associated with a severe prognosis of LPI [6]. Different pathways [7] may contribute to the development of surfactant accumulation within the alveoli in PAP resulting in decreased surfactant clearance mainly due to alterations in the phagocytic capacity of alveolar macrophages [8]. Practically, PAPs can be categorized into two types: autoimmune PAP and non-autoimmune PAP. The autoimmune PAP, accounting for 90% of cases, occurs more frequently in adulthood and is caused by disrupted GM-CSF signaling by neutralizing GM-CSF autoantibodies in the vast majority of cases [9]. . Nevertheless, pediatric patients are affected more often by non-autoimmune PAP, which can be of genetic origin (such as mutations of surfactant proteins), or secondary to a hematological or systemic disease (such as LPI) or toxic inhalation [10]. Based on the etiology, treatment of PAP most commonly consists of whole-lung lavage (WLL), in certain cases granulocyte-macrophage colony stimulating factor (GM-CSF) administration, and eventually lung transplantation [8]. While in autoimmune PAP there are anti GM-CSF antibodies and therefore GM-CSF administration is a promising pharmacotherapeutic approach [11]; research in LPI could not justify the role of GM-CSF administration since in LPI GM-CSF signaling pathway was found to be unaltered [12]. Nevertheless, recent case reports surprisingly observed a favorable response to GM-CSF therapy in patients with LPI-associated PAP as well [13–15].

Here we present a case of an 8-month-old infant presenting with respiratory failure caused by LPI-associated PAP. The infant was successfully treated with WLL on two occasions; first on veno-venous ECMO (VV ECMO) and then with the use of a selective bronchial blocker. After stabilization with WLL, she was successfully weaned from daytime respiratory support while first receiving subcutaneous and then inhaled GM-CSF therapy. This case gives further support to the notion that GM-CSF might be of benefit in patients with LPI-associated PAP.

## Case presentation

**Fig. 1:**
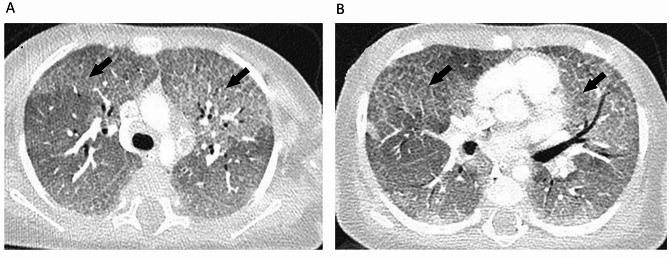
A CT scan of the chest revealed bilateral, diffuse ground-glass opacities with crazy paving pattern (arrows)

An 8-month-old female infant born as the first child of a nonconsanguineous Hungarian couple presented with severe respiratory failure with low oxygen saturation (70%) requiring intubation and mechanical ventilation with pressures of 16/5 cmH2O and 50% fraction of inspired oxygen. Chest radiograph at presentation revealed bilateral diffuse reticulogranular opacity. The infant was born at term gestation without complications and had been previously fit and well, with no history of immunocompromise, previous hospital admission or relevant family history. On examination the patient was pale and her weight was < 3 percentile (6600 g on admission). Hepatomegaly and splenomegaly were noted. As polymerase chain reaction test revealed Cytomegalovirus in the bronchoalveolar lavage, she was started on Valgancyclovir. Initial laboratory test results showed a white blood cell count of 11.85 × 10^9^/L, C-reactive protein of 3.7 mg/L, procalcitonin of 0.11 µg/L, LDH of 807 U/L, and serum ferritin of 707 µg/L. HIV test was negative and the lymphocyte subsets were normal. Immunoglobulin levels revealed low IgA concentration (0.57 g/L). Computed tomography (CT) scan of chest revealed a „crazy paving” pattern suggesting the diagnosis of PAP (Fig. 1). An empirical course of high dose corticosteroid therapy (5 mg/kg/day), azithromycin (10 mg/kg/day) and hydroxychloroquine (6 mg/kg/day) did not improve her respiratory status. The patient rapidly deteriorated and required high ventilatory support with 100% oxygen. Whole exome sequencing confirmed that the index patient was compound heterozygous for the maternal c.1429 + 2T > C and for the paternal c.821 A > G *SLC7A7* mutations. In accordance with the clinical presentation, *SLC7A7* mutations are responsible for LPI. Based on the genetic findings, our patient was started on a low protein intake diet with lysine, ornithine and citrulline supplementation. Neurodevelopmental examination was appropriate for age except for a 3-month delay in gross motor development. The parents underwent several multidisciplinary consultations where necessity for prolonged pediatric intensive care was repeatedly emphasized.

**Fig. 2:**
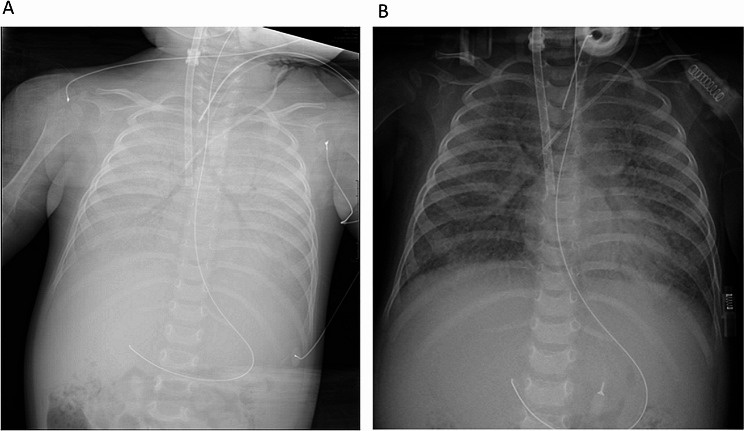
Chest X-ray prior **(A)** and after **(B)** the first whole lung lavage. Note the diffuse reticulogranular opacity **(A)**

At 10 month-of-age, the patient received tracheostomy for chronic ventilation. For children with PAP with moderate to severe respiratory symptoms, the treatment of choice is WLL [8, 16, 17]. Therefore, WLL was first attempted under general anesthesia and mechanical ventilation using a flexible fiberoptic bronchoscope. However, as the patient rapidly desaturated, the procedure was abandoned. During the ensuing multidisciplinary team meeting the decision was made to attempt the pulmonary lavage on VV ECMO support (Fig. 2). After percutaneous cannulation and the placement of the patient on ECMO support, the WLL was successfully performed with instillation of 25 mL of 37 °C normal saline in each lung at a time. In addition, WLL was performed with gentle suctioning using a syringe, because positioning the patient in the Trendelenburg and anti-Trendelenburg positions did not result in adequate draining of the saline from the lungs. The fluid collected was initially pink and turbid but it cleared up with time (Additional file 1, Fig. 1). The left and right lungs were rinsed with a total of 3100 mL warm saline over the course of the 210-minute procedure. Five days after the procedure, the infant was successfully weaned from ECMO and her respiratory status improved. The patient had been stable on CPAP provided through the tracheostomy for a few weeks. Head MRI revealed a small, subacute-appearing ischemic lesion in the left cerebellum. However, due to the gradual recurrence of respiratory deterioration one month after the first procedure, the pulmonary lavage was repeated under general anesthesia on mechanical ventilation using a selective bronchial blocker. The patient tolerated the procedure well and she was weaned to CPAP on the following day.

Simultaneously, at 11 month-of-age, the patient was started on regular subcutaneous GM-CSF administration at a dose of 10 µg/kg/day. During the subcutaneous GM-CSF administrations, she developed several episodes of fever and leukocytosis (peak leukocyte count of 20 × 10^9^/L) without identifiable infectious etiology. Her respiratory status also improved on CPAP and she was weaned to low-flow oxygen supplementation through the tracheal cannula and was transferred to the Pulmonology Unit. Two months later she was transferred to a local hospital requiring BiPAP noninvasive ventilation at night, while on low-flow oxygen support during daytime. At 22 months of age, she was switched to inhaled GM-CSF (sargramostin, 125 µg/day) administration followed by the cessation of recurrent fever and leukocytosis episodes. Subsequently, we were able to completely wean her from daytime respiratory support.

## Discussion and conclusions

We present a case of an 8-month-old infant presenting with respiratory failure due to LPI-associated PAP, whom we successfully weaned from daytime respiratory support while receiving initially subcutaneous then inhaled GM-CSF therapy after two courses of WLL. Follow-up at two years of age revealed normal cognitive function and speech development, and, not unexpectedly, a one-year delay in motor development.

Whole lung lavage is the gold standard therapy for PAP since the early 1960s [17], with positive response rates of 60–84% [18]. The procedure removes the lipoproteinaceous material from the alveoli [17]. However, the procedure is difficult to perform, is expensive and is not well tolerated by children [19]. Our patient’s course confirmed the challenges WLL therapy presents. Initially the patient was unstable and did not tolerate WLL while on mechanical ventilation. Therefore, the decision was made to place her on VV ECMO to increase the chance of success. In addition, her vessels were relatively gracile and difficult to cannulate. Veno-venous ECMO modality was chosen as her cardiac function was normal and thus only pulmonary bypass was needed. Also, VV ECMO has been associated with lower rate of complications compared to venoarterial ECMO [20]. Finally, even though WLL is classically performed through gravity with the patient being positioned in the reverse Trendelenburg and Trendelenburg positions, we were unable to drain the injected lavage fluid from the lungs by positioning alone. Therefore, we applied gentle airway suctioning using a syringe. Importantly, no complications occurred during the procedure and following the second round of WLL which was tolerated off VV ECMO, the patient could be weaned to non-invasive ventilation one day after the procedure.

A number of studies have evaluated the effect of the use of GM-CSF in autoimmune PAP. In cases of autoimmune PAP, lipids and proteins accumulate within the alveoli since alveolar macrophages are unable to catabolize surfactants due to disrupted GM-CSF signaling by neutralizing GM-CSF autoantibodies [9]. Granulocyte–macrophage colony-stimulating factor enhances the ability of macrophages to perform this function; therefore, GM-CSF replacement improves PAP pathology [21–23]. However, in non-autoimmune PAP, there is no rationale based on current science for the use of GM-CSF. Specifically, in LPI Barelli et al. found an unaltered GM-CSF signaling pathway, and since LPI patients did not have high levels of ant-GM-CSF antibodies, the impaired function and phagocytic activity of the macrophages was attributed to be intrinsic to the *SCL7A7* phenotype [12]. Surprisingly, recent case reports observed the resolution of PAP using GM-CSF in patients with LPI-associated PAP as well [13–15]. The rationale of this phenomenon is unknown, but the increase in the number and the activity of alveolar macrophages in the alveolar fluid by GM-CSF might be a reasonable explanation [13]. Additional hypotheses include that GM-CSF therapy might play a role in increasing in vivo gene expression involved in surfactant catabolism, and also that GM-CSF-dependent increase of *SLC7A7* mRNA might increase y + LAT1 protein that ameliorates macrophage function [15].

The deterioration of our patient to maximal conventional therapy after an empirical course of high dose corticosteroid, azithromycin and hydroxychloroquine, and the PAP reoccurrence after the first WLL prompted us to consider GM-CSF administration after the second WLL, based on the beneficial effect of GM-CSF in LPI-associated PAP available in recent case reports [13–15]. Initially, GM-CSF was administered subcutaneously and then the drug was switched to the inhaled route of administration. Although subcutaneously administered GM-CSF was found to be effective in patients with autoimmune PAP, minor complications such as fever, fatigue, headache, and injection site complications have been reported [24]. Our patient experienced fever and leukocytosis without proven infectious origin during the administration of subcutaneous GM-CSF. Meta-analyses suggest that, compared to subcutaneous GM-CSF therapy, inhaled GM-CSF is associated with a higher response rate, may act more peripherally and improves alveolar partial pressure of oxygen to a greater extent [24]. In agreement with these observations [24], our findings also suggest that compared to subcutaneous GM-CSF, inhaled GM-CSF might have been more beneficial in mucus mobilization and had fewer side effects. Importantly, it appears to have been more effective since we were able to completely wean the patient off respiratory support during daytime.

Of note is that, although rare, spontaneous remission of PAP might occur. Nevertheless, spontaneous remission of PAP is described mainly in autoimmune PAP, accounting for 5–10% of cases [9]. On the other hand, the early onset of pulmonary disease in LPI is associated with a severe prognosis [25], and specifically the early development of PAP was found to be significantly associated with death in a retrospective study investigating the long-term management of LPI [6]. It is therefore logical to assume that the marked improvement in our patient’s clinical course is attributed to the completion of the two WLLs and to the introduction of the GM-CSF therapy. This case alongside with the recently published case reports [13–15], gives further support to the notion that GM-CSF therapy might be of benefit in patients with LPI-associated PAP. Additional studies are needed to clarify the exact mechanism of resolution of PAP using GM-CSF in patients with LPI-associated PAP.

In conclusion, our case gives support to the recently published case reports that GM-CSF therapy might be of benefit in the treatment of LPI-associated PAP, and might represent a long-term therapeutic option following initial induction of remission with WLL. Further research is warranted to elucidate the exact therapeutic mechanism of GM-CSF therapy in patients with LPI-associated PAP.

### Electronic supplementary material

Below is the link to the electronic supplementary material.

Additional File 1, Fig.1.: Surfactant collected from the lungs of the index patient. The fluid collected was initially pink and dense, and it progressively became clearer.

## Data Availability

Further data that support the findings of this study are available upon reasonable request from the corresponding author.

## Abbreviations

LPI: lysinuric protein intolerance
PAP: pulmonary alveolar proteinosis
WLL: whole lung lavage
GM-CSF: granulocyte-macrophage colony stimulating factor
ECMO: extracorporal membrane oxygenation
VV ECMO: veno-venous extracorporal membrane oxygenation
