# Supplementary figures and images for: Whole lung lavage and GM-CSF use for pulmonary alveolar proteinosis in an infant with lysinuric protein intolerance: a case report
Source: Ital J Pediatr. 2024 Jun 3;50:111. doi: 10.1186/s13052-024-01677-y (PMC11149197; doi:10.1186/s13052-024-01677-y)

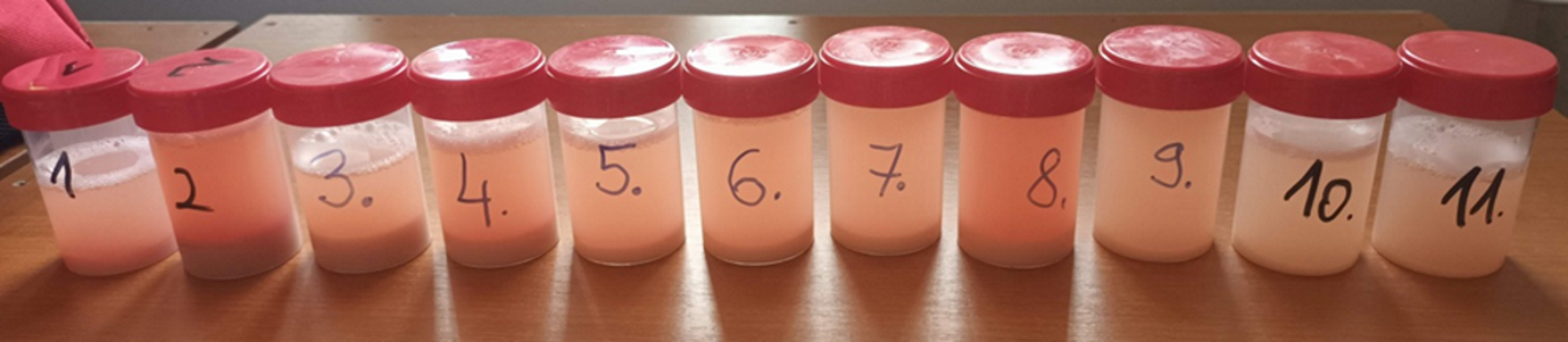

Supplement: Supplementary file 1 — Additional File 1, Fig.1.: Surfactant collected from the lungs of the index patient. The fluid collected was initially pink and dense, and it progressively became clearer. [file 13052_2024_1677_MOESM1_ESM.tiff]
